# Who could benefit the most from using a computer-aided detection system in full-field digital mammography?

**DOI:** 10.1186/1477-7819-12-168

**Published:** 2014-05-29

**Authors:** Na Young Jung, Bong Joo Kang, Hyeon Sook Kim, Eun Suk Cha, Jae Hee Lee, Chang Suk Park, In Young Whang, Sung Hun Kim, Yeong Yi An, Jae Jeong Choi

**Affiliations:** 1 Department of Radiology, Bucheon St. Mary’s Hospital, College of Medicine, The Catholic University of Korea, 327 Sosa-ro, Wonmi-gu, Bucheon-si, Gyeonggi-do 420-717, South Korea; 2 Department of Radiology, Seoul St. Mary’s Hospital, College of Medicine, The Catholic University of Korea, 222 Banpo-daero, Seocho-gu, Seoul 137-701, South Korea; 3 Department of Radiology, St. Paul’s Hospital, College of Medicine, The Catholic University of Korea, 180 Wangsan-ro, Dongdaemun-gu, Seoul 130-709, Korea; 4 Department of Radiology, Ewha Womans Mokdong Hospital, School of Medicine, Ewha Womans University, 1071 Anyangcheon-ro, Yangcheon-gu, Seoul 158-710, South Korea; 5 Department of Radiology, Human Medical Imaging and Intervention Center, 621 Gangnam-daero, Seocho-gu, Seoul 137-902, South Korea; 6 Department of Radiology, Incheon St. Mary’s Hospital, College of Medicine, The Catholic University of Korea, 56 Dongsu-ro, Bupyeong-gu, Incheon 403-720, South Korea; 7 Department of Radiology, Uijongbu St. Mary’s Hospital, College of Medicine, The Catholic University of Korea, 271 Cheonbo-ro, Uijeongbu-si, Gyeonggi-do 480-717, South Korea; 8 Department of Radiology, St. Vincent’s Hospital, College of Medicine, The Catholic University of Korea, 93 Jungbu-daero, Paldal-gu, Suwon-si, Gyeonggi-do 442-723, South Korea; 9 Department of Radiology, School of Medicine, Hallym University Medical Center, 7 Keunjaebong-gil, Hwaseong-si, Gyeonggi-do 445-170, South Korea

**Keywords:** Computer-aided detection (CAD), Mammography, Screening

## Abstract

**Background:**

The computer-aided detection (CAD) system on mammography has the potential to assist radiologists in breast cancer screening. The purpose of this study is to evaluate the diagnostic performance of the CAD system in full-field digital mammography for detecting breast cancer when used by dedicated breast radiologist (BR) and radiology resident (RR), and to reveal who could benefit the most from a CAD application.

**Methods:**

We retrospectively chose 100 image sets from mammographies performed with CAD between June 2008 and June 2010. Thirty masses (15 benign and 15 malignant), 30 microcalcifications (15 benign and 15 malignant), and 40 normal mammography images were included. The participating radiologists consisted of 7 BRs and 13 RRs. We calculated the sensitivity, specificity, positive predictive value (PPV) and negative predictive value (NPV) for total, normal plus microcalcification and normal plus mass both with and without CAD use for each reader. We compared the diagnostic performance values obtained with and without CAD use for the BR and RR groups, respectively. The reading time reviewing one set of 100 images and time reduction with CAD use for the BR and RR groups were also evaluated.

**Results:**

The diagnostic performance was generally higher in the BR group than in the RR group. Sensitivity improved with CAD use in the BR and RR groups (from 81.10 to 84.29% for BR; 75.38 to 77.95% for RR). A tendency for improvement in all diagnostic performance values was observed in the BR group, whereas in the RR group, sensitivity improved but specificity, PPV, and NPV did not. None of the diagnostic performance parameters were significantly different. The mean reading time was shortened with CAD use in both the BR and RR groups (111.6 minutes to 94.3 minutes for BR; 135.5 minutes to 109.8 minutes for RR). The mean time reduction was higher for the RR than that in the BR group.

**Conclusions:**

CAD was helpful for dedicated BRs to improve their diagnostic performance and for RRs to improve the sensitivity in a screening setting. CAD could be essential for radiologists by decreasing reading time without decreasing diagnostic performance.

## Background

The mammography is the single most effective method for screening breast cancer and can reduce breast cancer mortality [1,2]. However, the interpretation of screening mammography is challenging. The most significant limitation of screening mammography may be the false negative rate of between 10 and 25%. Many false negative interpretations are due to the interpretation of a large volume of images in order to detect a small number of cancers, the complex radiographic structure of the breast, the subtle mammographic findings of early breast cancer, and radiologist fatigue or distraction [2,3]. A large portion of breast cancers might be missed by the interpreting radiologist, even if they are experienced, but are frequently visible on previous mammograms [4,5].

To overcome the limitations of human observers and reduce the false negative rate of screening mammograms, double reading by another radiologist has been implemented at many hospitals. The results of studies indicate a potential 4 to 15% increase in the number of cancers detected as a result of double reading [6,7]. However, double reading cannot be widely adopted due to cost-effectiveness and practicality in most countries [8]. Thus, computer-aided detection (CAD) is widely used as a good alternative to double reading [2]. Many studies have revealed that CAD can reduce the false negative rate and increase the detection of breast cancer, particularly early breast cancer [2,3,8-10] without a significant increase in recall rate [2,8] and false positive rate for biopsy [8,10]. More recent studies have reported that CAD systems for full-field digital mammograms can also improve the diagnostic performance of mammograms [11,12]. Yang *et al.* reported that the CAD system can correctly mark most asymptomatic breast cancers detected with digital mammographic screening [11], and Bolivar *et al.* demonstrated that improved CAD sensitivity was maintained for small lesions and invasive lobular carcinomas, which have lower mammographic sensitivity [12].

CAD systems have the potential to assist both expert breast radiologists and community radiologists in the interpretation of mammograms, with larger improvements observed in community radiologists [13]. Several studies have demonstrated that the CAD application improves the diagnostic performance of non-expert radiologists [13-15]. The main advantage of CAD lies in the decreased false negative rate and improved sensitivity, regardless of radiologist experience. Therefore, the purpose of this study was to evaluate the diagnostic performance of a CAD system in full-field digital mammography for breast cancer detection when used by dedicated breast radiologist (BR) and radiology resident (RR), and to reveal who could benefit the most from CAD application.

## Methods

Institutional review board approval was received and informed consent was waived for this study. We retrospectively chose 100 image sets among mammographies performed between June 2008 and June 2010. All mammography examinations were performed with a digital mammography system (Selenia, Hologic: Bedford, Massachusetts, United States). Thirty masses (15 benign and 15 malignant), 30 microcalcifications (15 benign and 15 malignant), and 40 normal mammography images were included. A normal mammography was defined as images without initial significant findings and negative follow-up for at least two years. The exclusion criteria were: patients without biopsy results despite suspicious malignant findings, and patients without biopsy results who did not get a two-year follow-up mammography or sonography.

These mammography image sets consisted of a standard two view mammography, including craniocaudal and mediolateral oblique views. We obtained the compressed CAD images for review by Image Checker (R2, software; Los Altos, California, United States). The CAD information was presented to the radiologists as a low-resolution image embedded with marks. The mark for a mass was an asterisk, the mark for a microcalcification was a triangle, and the mark for a mass with microcalcification was a cross.

The participating radiologists consisted of 7 attending radiologists specializing in breast imaging (dedicated BRs), and 13 second- and third-year RRs. All BRs were board-certified radiologists who worked in a university-based breast imaging center. The mean experience period for BRs involving breast imaging was 9.9 years (between 4 and 16 years). Five radiologists in the BR group had previous experience with clinical CAD, but the remaining two did not. The second-year RRs had no previous experience with breast imaging, whereas the third-year RRs had at least four weeks of training experience in breast imaging. Before the first review we performed an educational lecture about CAD including CAD algorithms and various false positive marks for all radiologists involved in this study.

We arranged two different image sets with randomization. One set consisted of mammography images with CAD information for some cases and mammography images without CAD information for the others. The other set consisted of images with inversion of CAD assistance.

We randomized the order of the two image sets and had a washout period to minimize the memory of the previous evaluation. Each reviewer evaluated one image set and then reevaluated the other image set after at least one week washout period. The location (right or left, one of four quadrants), the type of the lesion (mass or microcalcification), and final assessment category were recorded by the reviewers for each case. Categorization was performed according to established guidelines [16]. The categories were: 1 for negative, 2 for benign, 3 for probably benign, 4 for suspicious lesion requiring biopsy and 5 for highly suggestive of malignancy. The 0 category was not used in this study. If there was more than one lesion in a patient, the reviewers chose the highest category for the most suspicious lesion. We checked the reading time during the two sets of categorization for each reviewer.

Sensitivity, specificity, positive predictive value (PPV) and negative predictive value (NPV) for total (n = 100), normal plus microcalcification (n = 70), and normal plus mass (n = 70), both with and without CAD use were calculated for each reviewer. We calculated and compared the mean and standard deviation of these diagnostic performance values with and without CAD use for the BR and RR groups. We also compared the mean diagnostic performance values in the BR group, according to the year of experience with breast imaging and previous clinical experience with CAD. We compared the diagnostic performance values for the RR group according to the training experience in breast imaging. The mean and standard deviation of reading time and time reduction for the BR and RR groups were obtained. Statistical analyses were performed with the chi-square test and Fisher’s exact test using SAS System for Windows V 9.1 (SAS Institute, Cary, North Carolina, United States). *P* <0.05 was considered significant.

## Results

Table 1 showed the mean and standard deviation of the diagnostic performance values with and without CAD use for the BR group, RR group, subgroups of the BR group according to the years of experience with breast imaging and previous clinical experience with CAD, and subgroups of the RR group according to training experience in breast imaging. Diagnostic performance was generally higher in the BR group than that in the RR group. Sensitivity improved with CAD use in both the BR and RR groups (81.10 ± 6.52 to 84.29 ± 4.18% for BR; 75.38 ± 13.8 to 77.95 ± 11.83% for RR). A tendency for CAD to improve all diagnostic performance values was observed in the BR group, whereas in the RR group sensitivity improved but specificity, PPV, and NPV did not. None of the diagnostic performance values showed significant differences.

**Table 1:** Diagnostic performance of mammography and mammography with computer-aided detection (CAD) for all cases (n = 100)

**Readers**	**Sensitivity**		**Specificity**		**PPV**		**NPV**	
**Total (n = 20)**	**Mammography**	**Mammo + CAD**	**Mammography**	**Mammo + CAD**	**Mammography**	**Mammo + CAD**	**Mammography**	**Mammo + CAD**
BR (n = 7)	81.10 ± 6.52	84.29 ± 4.18	70.82 ± 10.91	73.27 ± 10.32	56.30 ± 8.34	58.74 ± 8.31	90.87 ± 2.37	91.59 ± 1.63
RR (n = 13)	75.38 ± 13.85	77.95 ± 11.83	73.30 ± 10.66	69.56 ± 13.37	55.91 ± 7.47	54.13 ± 10.39	88.14 ± 5.55	88.03 ± 5.69
**BR subgroups**								
Ex ≥10* (n = 3)	83.33 ± 3.34	86.67 ± 0	74.76 ± 5.02	75.24 ± 2.97	58.85 ± 4.63	60.09 ± 2.95	91.30 ± 1.46	92.94 ± 0.25
Ex <10* (n = 4)	79.42 ± 8.29	82.50 ± 5.00	67.85 ± 13.92	71.79 ± 14.16	54.38 ± 10.65	57.72 ± 11.36	90.54 ± 3.08	90.59 ± 1.45
CAD ex (n = 5)	80.87 ± 7.88	83.34 ± 4.71	74.57 ± 5.84	77.43 ± 5.83	59.19 ± 5.93	61.86 ± 6.11	91.85 ± 1.86	91.64 ± 1.79
no CAD ex (n = 2)	81.67 ± 2.35	86.67 ± 0	61.43 ± 18.19	62.86 ± 14.14	49.06 ± 11.43	50.93 ± 9.69	88.42 ± 1.75	91.49 ± 1.76
**RR subgroups**								
RR(non-trained) (n = 6)	75.55 ± 15	75.55 ± 8.86	76.19 ± 12.21	70.00 ± 17.00	59.19 ± 9.46	54.62 ± 14.26	88.54 ± 5.95	86.32 ± 5.87
RR(trained) (n = 7)	75.24 ± 13.99	80.00 ± 14.27	70.82 ± 9.36	69.18 ± 10.78	53.11 ± 4.13	53.70 ± 6.78	87.80 ± 5.65	89.50 ± 5.52

Note.―All data are the percentages.

*Ex ≥10 means that the reviewer’s experience with breast imaging equal to or more than 10 years, while Ex <10 means less than 10 years.

BR, dedicated breast radiologists; ex, experience; mammo, mammography; NPV, negative predictive value; PPV, positive predictive value; RR, radiology residents.

The diagnostic performance was generally higher in the radiologists with more than 10 years’ experience than those with less than 10 years’ experience. The diagnostic performance showed a tendency to improve with the use of CAD in both experience groups. The diagnostic performance also improved with the use of CAD in groups with prior clinical CAD experience and those without CAD experience.

Diagnostic performance was not significantly different between the non-trained and one- month-trained RR groups. However, a sensitivity-improving tendency with CAD assistance was demonstrated only for the one-month-trained RR group and not for the non-trained RR group.

**Figure 1:**
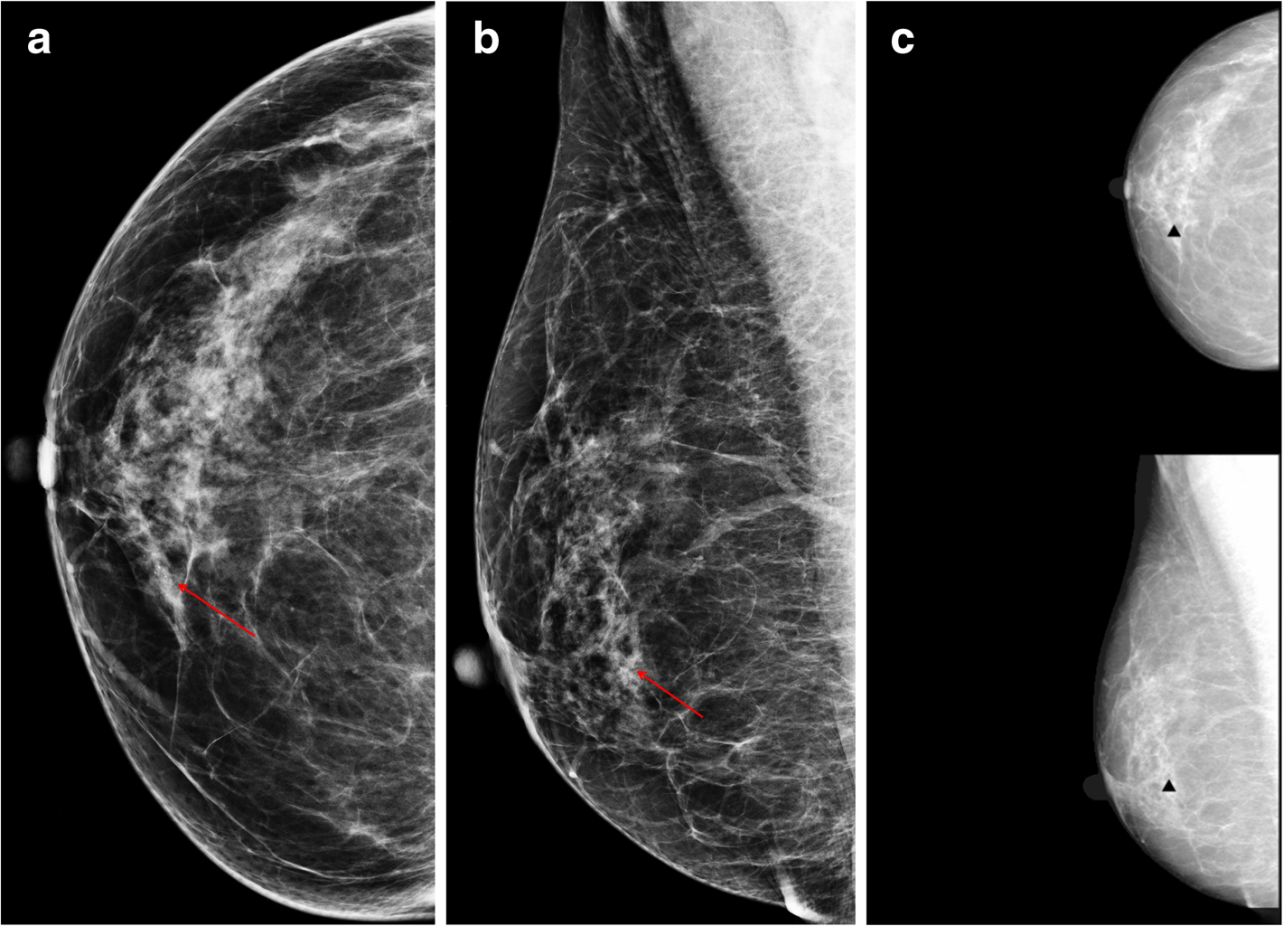
**A 53-year-old woman with ductal carcinoma *in situ *in the right breast.** The craniocaudal **(a)** and mediolateal oblique **(b)** views show the malignant microcalcifications (arrows) in the right lower inner quadrant. **(c)** The computer-aided detection (CAD) system correctly marked the microcalcifications (triangles), which improved sensitivity.

**Figure 2:**
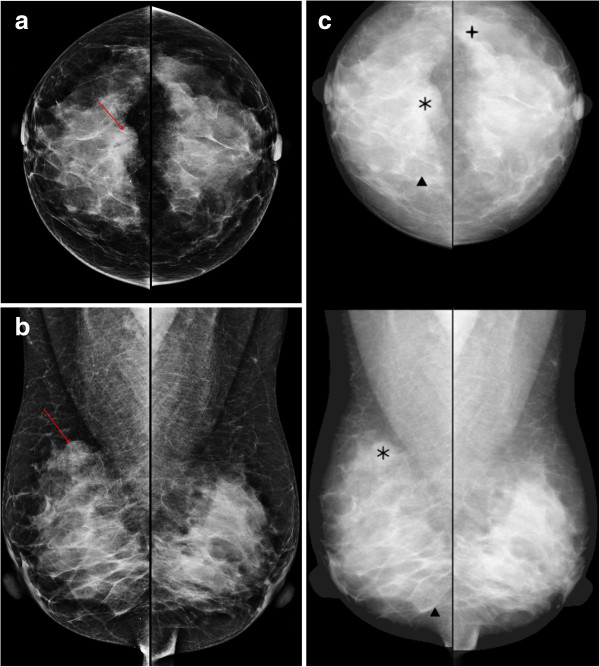
**A 49-year-old woman with invasive ductal carcinoma in the right breast.** The craniocaudal **(a)** and mediolateal oblique **(b)** views show the partly circumscribed mass (arrows) in the right mid upper breast. **(c)** The computer-aided detection (CAD) system correctly marked the mass (asterisks), which improved sensitivity.

When the data were evaluated separately for the two case groups (normal plus microcalcification versus normal plus mass), diagnostic performance was generally higher for the microcalcification cases than that for the mass cases (Figures 1 and 2). Tables 2 and 3 show the mean and standard deviation of the diagnostic performance values with and without CAD use in the normal plus microcalcification case group (Table 2) and the normal plus mass case group (Table 3) for each BR and RR group and subgroup. Sensitivity, specificity, PPV, and NPV in the BR group for the normal plus microcalcification cases increased slightly but not significantly with the use of CAD, however all these parameters remained unchanged or even slightly decreased in the RR group though it was not significant (Table 2). The results of masses varied in the BR and RR groups (Table 3).

**Table 2:** Diagnostic performance of mammography and mammography with computer-aided detection (CAD) for normal plus microcalcification cases (n = 70)

**Readers**	**Sensitivity**		**Specificity**		**PPV**		**NPV**	
**Total (n = 20)**	**Mammography**	**Mammo + CAD**	**Mammography**	**Mammo + CAD**	**Mammography**	**Mammo + CAD**	**Mammography**	**Mammo + CAD**
BR (n = 7)	93.33 ± 10.18	95.24 ± 5.04	75.59 ± 11.35	78.70 ± 11.10	52.96 ± 12.69	57.18 ± 11.89	97.29 ± 4.51	98.33 ± 1.75
RR (n = 13)	87.69 ± 13.29	87.69 ± 11.50	78.32 ± 7.62	73.71 ± 10.70	53.44 ± 8.40	49.46 ± 11.42	96.20 ± 3.98	95.56 ± 4.24
**BR subgroups**								
Ex ≥10* (n = 3)	97.78 ± 3.85	97.78 ± 3.85	80.61 ± 8.20	81.82 ± 5.46	59.14 ± 11.06	60.03 ± 7.45	99.26 ± 1.28	99.28 ± 1.25
Ex <10* (n = 4)	90.00 ± 12.77	93.33 ± 5.44	71.82 ± 12.98	76.36 ± 14.47	48.32 ± 13.18	55.05 ± 15.22	95.81 ± 5.72	97.62 ± 1.88
CAD ex (n = 5)	97.33 ± 5.96	96.00 ± 5.96	79.27 ± 6.37	82.54 ± 6.25	56.99 ± 9.17	61.02 ± 9.48	99.07 ± 2.08	98.72 ± 1.96
no CAD ex (n = 2)	83.33 ± 14.14	93.33 ± 0	66.37 ± 19.28	69.09 ± 18.00	42.87 ± 18.58	47.59 ± 15.20	92.83 ± 7.00	97.36 ± 0.67
**RR subgroups**								
RR(non-trained) (n = 6)	87.78 ± 12.23	84.44 ± 10.04	80.00 ± 8.83	74.24 ± 14.29	55.92 ± 10.82	50.37 ± 16.2	96.19 ± 3.63	94.13 ± 4.30
RR(trained) (n = 7)	87.62 ± 15.12	90.48 ± 12.68	76.88 ± 6.78	73.25 ± 7.62	51.32 ± 5.69	48.69 ± 6.39	96.21 ± 4.56	96.79 ± 4.08

Note.―All data are the percentages.

*Ex ≥10 means that the reviewer’s experience with breast imaging equal to or more than 10 years, while Ex <10 less than 10 years.

BR, dedicated breast radiologists; ex, experience; mammo, mammography; NPV, negative predictive value; PPV, positive predictive value; RR, radiology residents.

**Table 3:** Diagnostic performance of mammography and mammography with computer-aided detection (CAD) for normal plus mass cases (n = 70)

**Readers**	**Sensitivity**		**Specificity**		**PPV**		**NPV**	
**Total (n = 20)**	**Mammography**	**Mammo + CAD**	**Mammography**	**Mammo + CAD**	**Mammography**	**Mammo + CAD**	**Mammography**	**Mammo + CAD**
BR (n = 7)	74.28 ± 8.97	73.33 ± 6.67	79.22 ± 11.15	81.30 ± 9.72	51.63 ± 8.65	54.15 ± 10.37	92.12 ± 2.25	91.84 ± 1.3
RR (n = 13)	63.08 ± 17.77	68.21 ± 14.95	82.66 ± 9.92	80.00 ± 14.34	52.04 ± 10.23	53.70 ± 15.71	89.58 ± 4.16	90.25 ± 3.65
**BR subgroups**								
Ex ≥10* (n = 3)	68.89 ± 3.85	75.55 ± 3.85	84.85 ± 1.05	84.85 ± 2.10	55.37 ± 0.32	57.75 ± 3.10	90.92 ± 0.94	92.73 ± 1.01
Ex <10* (n = 4)	78.33 ± 10.00	71.67 ± 8.39	75.00 ± 13.88	78.64 ± 12.80	48.82 ± 11.18	51.45 ± 13.64	93.03 ± 2.64	91.17 ± 1.15
CAD ex (n = 5)	72.00 ± 2.98	70.66 ± 5.96	82.55 ± 5.69	84.73 ± 5.55	53.80 ± 6.92	57.11 ± 8.30	91.52 ± 0.83	91.42 ± 1.13
no CAD ex (n = 2)	80.00 ± 18.85	80.00 ± 0	70.91 ± 20.57	72.73 ± 15.43	46.2 ± 13.24	46.75 ± 14.69	93.63 ± 4.60	92.89 ± 1.41
**RR subgroups**								
RR(non-trained) (n = 6)	63.33 ± 20.55	66.67 ± 13.33	83.64 ± 11.03	77.27 ± 17.99	53.81 ± 13.48	50.1 ± 17.04	89.73 ± 4.84	89.25 ± 3.76
RR(trained) (n = 7)	62.86 ± 16.72	69.52 ± 17.15	81.82 ± 9.68	82.34 ± 11.29	50.52 ± 7.22	56.78 ± 15.08	89.44 ± 3.88	91.1 ± 3.61

Note.―All data are the percentages.

*Ex ≥10 means that the reviewer’s experience with breast imaging equal to or more than 10 years, while Ex <10 less than 10 years.

BR, dedicated breast radiologists; ex, experience; mammo, mammography; NPV, negative predictive value; PPV, positive predictive value; RR, radiology residents.

The mean reading time decreased with the use of CAD in the BR and RR groups (127.1 ± 40.0 to 104.3 ± 34.2 minutes per image set for all; 111.6 ± 36.0 to 94.3 ± 26.1 minutes per image set for BR; 135.5 ± 40.2 to 109.8 ± 37.8 minutes per image set for RR) (Table 4). The mean time reduction was higher for the RR group than that in the BR group (−17.2 ± 19.7% versus −12.6 ± 19.6%) and was more noticeable in the one-month-trained RR group (−24.0 ± 21.0%).

**Table 4:** Reading time for mammography and mammography with computer-aided detection (CAD)

**Readers**	**Method**	**Reading time**		**Time reduction**	
		**Mean ± SD**	**Median (min-max)**	**Mean ± SD (%)**	**Median (min-max) (%)**
**BR + RR (n = 20)**	**mammo**	127.1 ± 40.0	120.0(68.0-210.0)	-	-
	**mammo + CAD**	104.3 ± 34.2	101.5(64.0-210.0)	−15.6 ± 19.2	−16.0(−46.7-29.4)
**BR (n = 7)**	**mammo**	111.6 ± 36.0	103.0(68.0-160.0)	-	-
	**mammo + CAD**	94.3 ± 26.1	88.0(64.0-140.0)	−12.6 ± 19.6	−15.4(−30.0-29.4)
**RR (n = 13)**	**mammo**	135.5 ± 40.2	120.0(70.0-210.0)	-	-
	**mammo + CAD**	109.8 ± 37.8	103.0(66.0-210.0)	−17.2 ± 19.7	−16.7(−46.7-13.0)
**Non-trained RR (n = 6)**	**mammo**	145.8 ± 36.4	145.0(100.0-200.0)	-	-
	**mammo + CAD**	132.7 ± 41.9	116.5(100.0-210.0)	−8.8 ± 15.4	−11.1(−28.6-13.0)
**Trained RR (n = 7)**	**mammo**	126.6 ± 43.9	120.0(70.0-210.0)	-	-
	**mammo + CAD**	90.1 ± 20.4	85.0(66.0-120.0)	−24.4 ± 21.0	−26.1(−46.7-8.9)

Note.―The reading time was shown in minutes and time reduction in percentage.

BR, dedicated breast radiologists; mammo, mammography; RR, radiology residents.

## Discussion

Improved breast cancer detection has been demonstrated using the CAD system in many studies [2,3,8-10]. Some prospective studies have shown that CAD improves cancer detection rate by between 4.7 and 19.5% [3,8,10,17]. Previous studies have also shown that CAD increases diagnostic performance, particularly sensitivity [12,18]. In our study, sensitivity tended to improve with the use of CAD for the BR and RR groups.

CAD systems were initially designed to detect potential malignancies in the breast, so the detection algorithms were heavily biased towards sensitivity, thereby sacrificing the specificity of any mark [8]. Few studies have evaluated the specificity of the CAD system and the reported specificity after applying CAD either decreases or remain unchanged [10,19]. Singh *et al.*[20] reported that both sensitivity and specificity improved for expert radiologists and residents using their CAD model, the so-called linear discriminant analysis. However this model included not only mammographic, but also ultrasonographic features, which improved specificity in that study. In our study, it was promising that specificity in the BR group increased for total and normal plus microcalcification cases without a decrease in sensitivity.

Several studies have shown that CAD improves the diagnostic performance of non-expert radiologists [13-15] or even students [15]. CAD systems assist both expert breast radiologists and community radiologists in interpreting mammograms, with a larger improvement in community radiologists [13]. A study by Quek *et al.*[14] demonstrated that the CAD system significantly improves the detection of suspicious mammographic abnormalities by inexperienced radiologists. In another study, Sohns *et al.* performed a receiver-operating characteristics analysis and showed that the greatest benefit after the use of CAD was observed for students, followed by residents and mammography fellows [15]. This outcome showed that the maximal CAD effect was even greater if the readers’ experience was lower. In our study, sensitivity, specificity, PPV, and NPV tended to increase with CAD use in the BR group, whereas only sensitivity increased slightly in the RR group. The experienced dedicated breast radiologist can take advantage of CAD more efficiently than radiology residents with less experience in breast imaging. Experience with breast imaging had a more important effect on the improvement of diagnostic performance with CAD application than previous CAD experience. The previous one-month training experience of radiology residents had a favorable effect on improved sensitivity with CAD use. The improvement in specificity with CAD assistance was not demonstrated for the non-experienced or less-experienced radiologist in our study, therefore it can have limited value and even be harmful for a non-expert radiologist to use the CAD system for a correct diagnosis. However, the improvement in the sensitivity of mammograms is the more important purpose of CAD, particularly for less experienced readers.

The sensitivity of CAD for microcalcifications is generally higher than that for masses [12,18]. In our study, the diagnostic performance for microcalcifications was generally higher than that for masses in both the BR and RR groups. No significant difference in diagnostic performance was observed in either the BR or RR groups when CAD was applied, but the results showed a tendency for an improvement in sensitivity, specificity, PPV, and NPV in the BR group when the radiologists evaluated microcalcifications but not masses. The sensitivity for microcalcification improved slightly with CAD use for the one-month-trained RR subgroup, but not for the non-trained RR subgroup. Figure 1 demonstrates the malignant microcalcification case with a true positive CAD marker, which turned out to be ductal carcinoma *in situ*. The CAD marker was helpful for some of the BRs and RRs to make a correct evaluation.The CAD marker for masses was also helpful for some radiologists to make the correct evaluation confidently. Figure 2 shows that the mass marker was of a benefit in detecting and diagnosing the invasive ductal carcinoma in the right breast.

**Figure 3:**
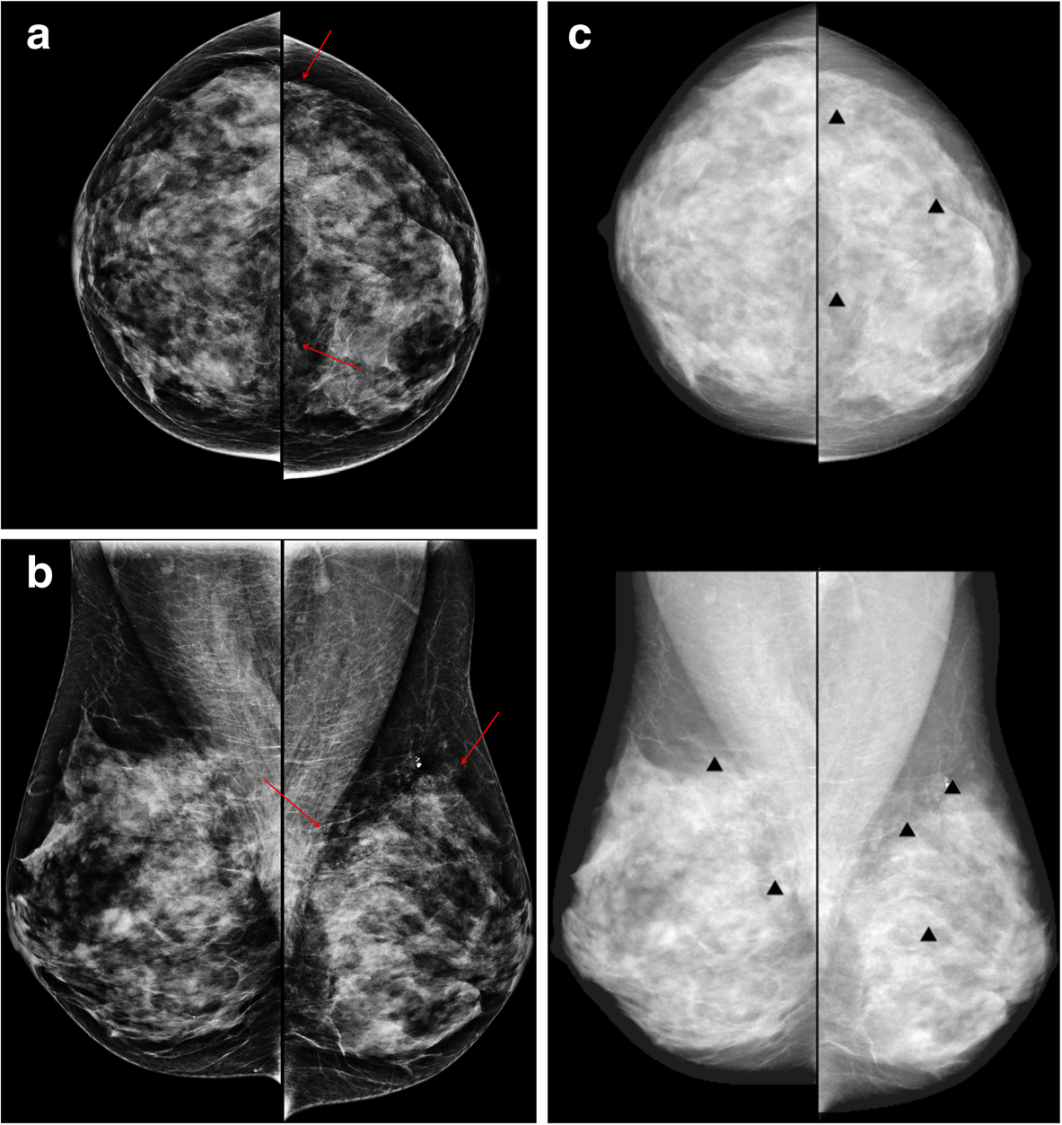
**A 50-year-old woman with fibrocystic changes in the left breast.** Mammography craniocaudal **(a)** and mediolateal oblique **(b)** views show regional punctate or milk-of-calcium microcalcifications (arrows) in the left upper outer and inner quadrant. **(c)** The computer-aided detection (CAD) system indicated several false-positive microcalcification marks (triangles) in the left breast.

One of the main disadvantages of CAD is the high rate of false positive marks. Figure 3 shows several false positive microcalcification marks in the left breast, resulting in a misdiagnosis of benign microcalcifications related to fibrocystic changes, which were read as malignant microcalcifications by some readers. The false positive marks distracted the radiologists and potentially elongated reading time. Sohns *et al.* reported a significant elongation of reading time with CAD use [15]. However, interpretation time increased slightly in another study about CAD usefulness [21] but was not significantly higher after an interactive CAD system was used (84.7 ± 61.5 seconds per case in an unaided session to 85.9 ± 57.8 seconds per case in a CAD-aided session, *P* = 0.13). In our study, a reduction of reading time was demonstrated for both groups. The mean time reduction was higher for the RR than the BR group. Approximately 71% (5 out of 7) of BRs and 46% (6 out of 13) of RRs participating in our study had previous experience with a clinical CAD system. We also conducted an educational lecture about CAD. The direct and indirect experience using CAD enabled the radiologists to discard most of false positive marks confidently without consuming time. The current CAD software versions were upgraded toward the acceptable false positive marks, which could also be helpful for reducing readers’ interpretation time.

There were some limitations to our study. First, only 100 randomly selected test cases were used for evaluation. The small number of cases may have prevented any significant differences in diagnostic performances with or without CAD use. Secondly, we included various sizes of microcalcifications and masses. Each reader could evaluate the different lesion with the highest suspicion. We tried to include just one lesion in each case, excluding the possibility of each reader evaluating different lesions. Finally, only 20 radiologists participated in this study as readers. However we included many radiologists with various amounts of experience and we evaluated which group of radiologists would benefit from using a CAD system.

## Conclusions

In conclusion, our results demonstrate that CAD was helpful for dedicated breast radiologists to improve diagnostic performance and for non-expert radiologists to improve sensitivity in a screening setting. A CAD system can contribute to shortening the reading time in both breast-dedicated radiologists and residents without decreasing diagnostic performance. However, CAD systems are especially useful for dedicated breast radiologists, therefore CAD systems used by non-expert radiologists can even be harmful when they reduce the time of detection but do not improve the specificity of the diagnosis. CAD could provide essential assistance to radiologists, especially dedicated breast radiologists, by decreasing reading time without decreasing diagnostic performance.

## Abbreviations

BR: Breast radiologists; CAD: Computer-aided detection; NPV: Negative predictive value; PPV: Positive predictive value; RR: Radiology residents.

## Competing interests

The authors declare that they have no competing interests.

## Authors’ contributions

NYJ and BJK participated in conception of the study, data collection and analysis, and drafted the manuscript. HSK, ESC, JHL, CSP, IYW, SHK, YYA and JJC participated in data collection and analysis. All authors read and approved the final manuscript.
